# TogoGenome/TogoStanza: modularized Semantic Web genome database

**DOI:** 10.1093/database/bay132

**Published:** 2019-01-08

**Authors:** Toshiaki Katayama, Shuichi Kawashima, Shinobu Okamoto, Yuki Moriya, Hirokazu Chiba, Yuki Naito, Takatomo Fujisawa, Hiroshi Mori, Toshihisa Takagi

**Affiliations:** 1 Database Center for Life Science, Joint Support-Center for Data Science Research, Research Organization of Information and Systems, Wakashiba, Kashiwa-shi, Chiba, Japan; 2 National Institute of Genetics, Mishima, Shizuoka, Japan; 3 Department of Biological Sciences, Graduate School of Science, The University of Tokyo, Yayoi, Bunkyo-ku, Tokyo, Japan

## Abstract

TogoGenome is a genome database that is purely based on the Semantic Web technology, which enables the integration of heterogeneous data and flexible semantic searches. All the information is stored as Resource Description Framework (RDF) data, and the reporting web pages are generated on the fly using SPARQL Protocol and RDF Query Language (SPARQL) queries. TogoGenome provides a semantic-faceted search system by gene functional annotation, taxonomy, phenotypes and environment based on the relevant ontologies. TogoGenome also serves as an interface to conduct semantic comparative genomics by which a user can observe pan-organism or organism-specific genes based on the functional aspect of gene annotations and the combinations of organisms from different taxa. The TogoGenome database exhibits a modularized structure, and each module in the report pages is separately served as TogoStanza, which is a generic framework for rendering an information block as IFRAME/Web Components, which can, unlike several other monolithic databases, also be reused to construct other databases. TogoGenome and TogoStanza have been under development since 2012 and are freely available along with their source codes on the GitHub repositories at https://github.com/togogenome/ and https://github.com/togostanza/, respectively, under the MIT license.

## Background

In life sciences, genome sequences have served as a central resource like a base map at which essential information, such as gene structures, regulatory regions, variations and their functional annotations, could be integrated. As genome projects are conducted on various species, the genomic sequences and gene annotations are deposited into a public database, the International Nucleotide Sequence Database Collaboration (INSDC) (1), which is jointly operated by the DNA Databank of Japan (DDBJ) (2), GenBank at National Center for Biotechnology Information (NCBI) (3) and European Nucleotide Archive (ENA) at The European Molecular Biology Laboratory - The European Bioinformatics Institute (EMBL-EBI) (4). However, each genome project often constructs its own genome database to add and update detailed annotations. For this purpose, generic and open source genome databases such as GMOD (5), Ensembl (6) and InterMine (7) can be used.

These major database systems serve genome annotations for a large number of species. However, because these genome databases have been monolithically constructed, it is difficult to reuse their components even though the represented information is very similar. Meanwhile, to extend a system that represents information unique to an organism, the inclusion of additional annotations may require to change the database schema and to make significant modifications to the system. In contrast, because any data can be expressed in a same format in Resource Description Framework (RDF), it is possible to easily integrate a wide variety of data from gene annotations to phenotypes and habitat environments of organisms. Also, there is no limit to the type of data that can be stored in an RDF database. Each piece of information integrated into the RDF is distinguished by a globally unique identifier in the form of Uniform Resource Identifier (URI); thus, the related information can be seamlessly linked and traced by the URIs.

Based on our experiences in the genome annotation and the construction of genome databases, we realized that most of the annotation information can be stylized. Therefore, it would be efficient to freely select the predefined modularized components for creating a genome database instance along with developing only new components based on annotations that are unique to the target organism stored as RDF data. Thus, it is expected that the cost required to construct a new genome database could be considerably reduced by managing all the annotation information in RDF and by providing the visualization modules for each subset of categorized annotations as reusable components. However, there was no precedent genome database that was purely based on RDF data; therefore, a demonstration was required to verify whether the use of SPARQL Protocol and RDF Query Language (SPARQL) would be practical and scalable enough for a genome database.

## Results

We developed a purely RDF-based genome database, TogoGenome, that was primarily based on the RefSeq (8) and the UniProt (9) data. UniProt has been publishing their data in RDF since 2008 (10); however, there has been no RDF representation of the RefSeq genome annotations. Therefore, in collaboration with DDBJ, we developed a converter of INSDC (DDBJ/GenBank/ENA) and RefSeq entries into RDF data. We have also developed ontologies for the INSDC-annotated sequences and taxonomy (http://ddbj.nig.ac.jp/ontologies/) as well as feature locations (see the Materials and Methods section).

TogoGenome uses the Semantic Web technology for data integration by which all the data are aggregated in RDF and semantically annotated with ontologies. Therefore, in addition to a basic keyword searches, faceted searches with various aspects based on the semantic hierarchy of the data can be performed. Further, all the RDF data can be freely accessed by SPARQL queries not only from a web interface but also from a program. Bioinformaticians can easily develop a program to acquire the data sets of necessity, perform analyses and develop their own summarizations and visualizations according to their requirements.

To produce reusable components, we developed the TogoStanza, which is a framework for visualizing the result of a SPARQL query as an IFRAME or as Web Components (https://www.webcomponents.org/), which can be embedded into any HTML web page. Any number of components can be freely chosen and combined to generate a resulting page, which could not have been easily realized using the monolithic databases. In fact, TogoGenome displays various search results as a report page by combining with the related TogoStanza in an arbitrary context such as a gene, an organism, a phenotype or an environment.

### TogoGenome

TogoGenome is a Semantic Web-based genome database in which heterogeneous information is compiled from various RDF data annotated with ontologies. With the RDF data and ontologies, TogoGenome provides several query interfaces. First, a user can conduct a faceted search based on a combination of gene, taxonomy, phenotype and environment ontologies. Second, a simple comparative genomic analysis can be performed among the genes of several species based on the common and unique gene annotations. Finally, as in the traditional genome databases, TogoGenome data can be searched using a free text keyword or a genomic sequence. However, dedicated text-indexing systems are required because a SPARQL query is not efficient enough to perform free text search (see the Materials and Methods section).

#### Ontology-based faceted search

One of the main interests of current biology is the relationships between genotypes and phenotypes. In case of humans, the most important relation is between genes and diseases. In case of crops and livestock, the genetic factors related to the aspects of quantity and quality, such as yield, nutritional value and gustation, are of considerable interest. In microorganisms, the effects of gene functions on the physiology, metabolites and interaction with the environment are typical examples of the subjects of research.

To elucidate these relations, a bioinformatics approach is required to efficiently formulate the hypotheses using the knowledge in the databases and to verify the hypotheses by performing experiments. However, the genomic and phenotypic information are scattered throughout the genes, pathways, literature databases and so on. There is no efficient database system to search for genes of various species in combination with the phenotypes.

**Figure 1:**
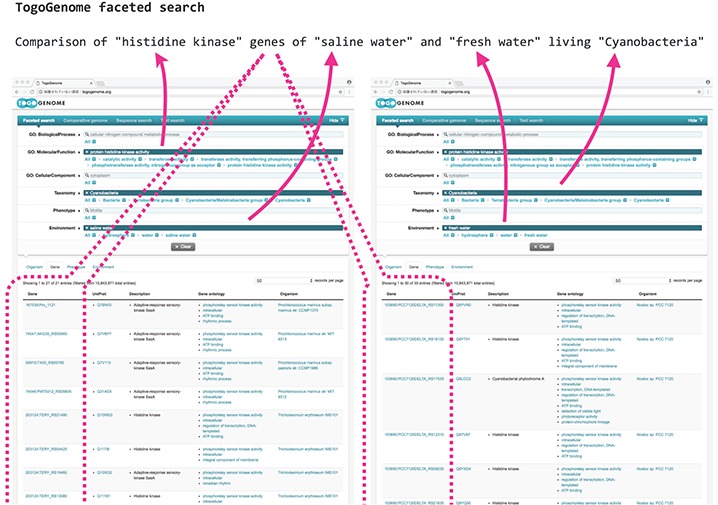
TogoGenome faceted search.

As an example, suppose if a scientist intended to verify the difference in the composition of the cyanobacterial gene sets related to environmental responses, such as ‘histidine kinases’, by comparing the gene sets of marine and freshwater living species, the scientist must narrow down those genes that have the desired function by (i) obtaining a list of cyanobacteria from the taxonomy database, (ii) selecting those species for which the complete genome has been decoded by searching the genome databases, (iii) identifying whether the growth environment of each cyanobacterium is seawater or freshwater using the literature and other databases, (iv) acquiring the gene set of each species and (v) obtaining annotations for each gene set with the help of a gene ontology (GO) to acquire the intended gene set. This procedure is difficult to automate; therefore, it was necessary for researchers to manually investigate each database.

TogoGenome provides an ontology-based faceted search interface to achieve this objective. A user can select ‘Cyanobacteria’ from ‘Taxonomy’, specify ‘protein histidine kinase activity’ from ‘GO: Molecular Function’ and select ‘saline water’ and ‘fresh water’ from ‘Environment’ to obtain the desired gene sets (Figure 1).

#### Semantic comparative genomics

Because UniProt proteins are semantically annotated in RDF and because TogoGenome holds the links between proteins and genes that are encoded in the genome of each organism, UniProt annotations can be used to find a specific subset of genes by selecting the attributes that are common or unique to a given set of species. First, a category of the annotation aspect can be selected from the protein motif, sub-cellular location, pathway, GO, enzyme classification or ortholog classifications. Second, a maximum of five species can be selected to compare the gene set. Third, a list of functional classifications that are common only to the selected combination of organisms is presented. Fourth, one of the objective classifications can be selected to obtain a corresponding list of genes in the target organisms.

**Figure 2:**
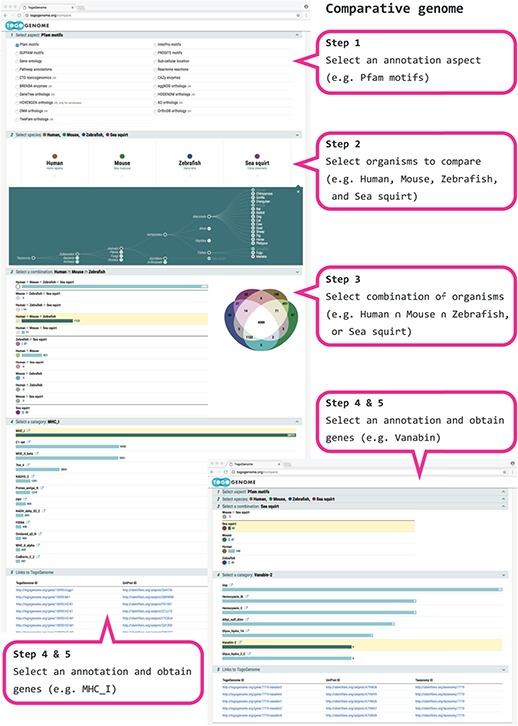
TogoGenome comparative genomics.

As an example, we (1) select the ‘Pfam motifs’ as an annotation aspect and (2) specify human, mouse, zebrafish and sea squirt as the target set of organisms to perform the comparison. Further, (3a) we select a combination of human ∩ mouse ∩ zebrafish and (4a) find the Major Histocompatibility Complex (MHC) domains corresponding to the adaptive immune system that are observed in vertebrates (therefore, not observed in sea squirts) (11) or (3b) select only the sea squirt and (4b) find Vanavin-2, which is a domain that is unique to sea squirts for oxygen binding (Figure 2).

#### Text index search

TogoGenome also provides simple keyword and sequence search interfaces. Because the text search function that is implemented in the existing RDF database is inadequate, we use Apache Solr (http://lucene.apache.org/solr/) to perform the keyword search and the GGGenome service to perform the sequence search (see the Materials and Methods section). While performing the keyword search, a list of TogoStanza, which contains the keywords, is presented on the basis of a free text match for gene names, species names, phenotype terms and environmental terms. In a sequence search, a list of reference genomes, which includes a specified sequence, is exhibited with links to the TogoGenome genes, which reside in the overlapping or surrounding regions of the query sequence in the genome.

### TogoStanza

Majority of the existing genome databases comprise typical components such as a gene name and aliases with a brief description, a chromosomal location and gene structures of the transcripts as a genome browser, the corresponding nucleotide sequences and amino acid sequences, the functional annotations of the genes and proteins, the sequence variations and modifications, the corresponding ortholog genes in other species, the relevant literature and cross-references to the external databases. Despite the fact that several pieces of information are commonly represented, they cannot be reused while developing a new database because most of the existing databases are monolithic. In fact, when our collaborator started to develop the MicrobeDB.jp (https://microbedb.jp/) and CyanoBase (12) databases, combining their original annotations with the existing information that was stored in the major genome databases was difficult; therefore, they were forced to develop their own genome databases from scratch even though some of the contents were imported from the existing databases. To overcome this limitation, we developed TogoStanza to enable database developers to reuse the components of the TogoGenome database in their genome databases. Because the TogoStanza system is designed to be generic, it is not limited to the genome databases and is being utilized in other domains, such as proteomics and glycomics databases, as well as some other web applications.

**Figure 3:**
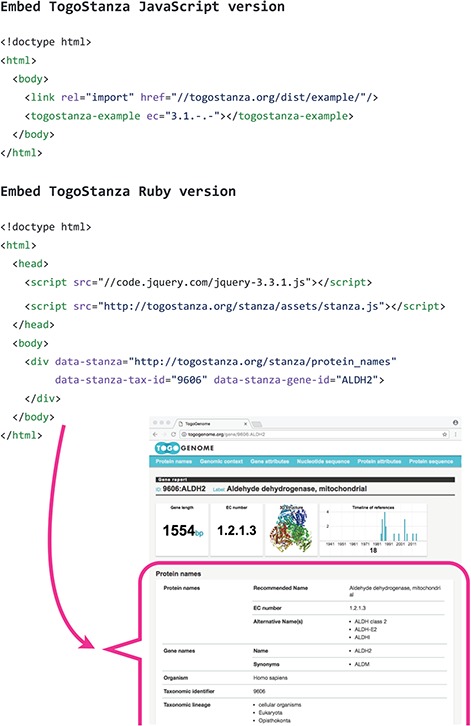
Embedding TogoStanza into a web page.

#### Features of the TogoStanza

**Figure 4:**
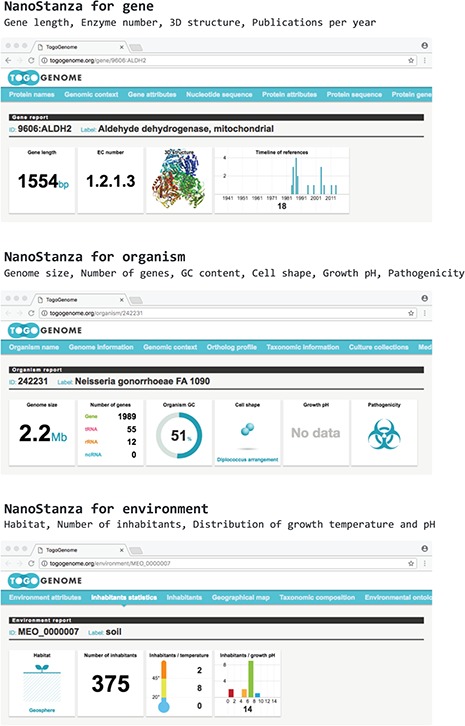
NanoStanza in gene, organism and environment report pages.

TogoStanza is a web application framework that obtains information from the web Application Programming Interface (API), SPARQL in particular, and visualizes the results as an IFRAME or Web Components that can be embedded into any web page (Figure 3). TogoGenome provides the report pages for each gene, organism, phenotype and environment. The pages display all the information by combining a series of related TogoStanza. In the case of the gene report page, each TogoStanza takes a taxonomy ID and gene ID as its arguments, obtains information related to the gene using dedicated SPARQL queries and visualizes the results in HTML. All technologies, such as HTTP, AJAX, HTML, CSS and JavaScript, are web standards so that any web application developer can easily create or customize a TogoStanza for publication online, even though optimizing the performance of a SPARQL query may require some specialized tuning techniques based on the domain knowledge and RDF data. A list of currently available TogoStanza used in the TogoGenome database can be found at http://togostanza.org/ where users can try out its functionality by changing the arguments on the fly. Additionally, NanoStanza is another form of TogoStanza that summarizes information at a glance in an icon-sized module (Figure 4). The metadata of each TogoStanza is written in the JSON-LD format and is used to automatically summarize and categorize each TogoStanza in the showcase page.

To date, more than 250 TogoStanza have been developed, including those developed for databases other than the TogoGenome database (Table 1). The TogoStanza framework is well suited to web application development, especially for the Semantic Web data in various life sciences and biomedical domains. In BioJS (13), which is a similar web application framework that is not specialized for the Semantic Web, 195 components are provided. Among these components, only one module (nextprot-cli) seems to use SPARQL.

Using TogoStanza, components that are common to several databases in life sciences and biomedical domains are successfully modularized, leading to a reduction in the development costs and making the resulting database to be extensible for new functionalities. Further, each distributed primary data provider can be responsible for the reuse of the latest database contents, which will be adequately credited in other databases.

**Table 1:** List of TogoStanza providers

**Database**	**Domain**	**Number of TogoStanza**	**URL**
TogoGenome	Genome	59	http://togogenome.org/stanza
MicrobeDB.jp	Genome	113	http://microbedb.jp/stanza/
CyanoBase	Genome	6	http://genome.microbedb.jp/stanza
MBGD	Ortholog	19	http://mbgd.genome.ad.jp/stanza/
GlyTouCan	Glycomics	16	https://bitbucket.org/glycosw/glytoucan-stanza https://github.com/glytoucan/glytoucan-js-stanza
jPOST	Proteomics	15	http://tools.jpostdb.org/ts/stanza/
TogoVar	Variation	26	https://togovar.biosciencedbc.jp/stanza

## Materials and methods

### Integration of genome annotations

**Figure 5:**
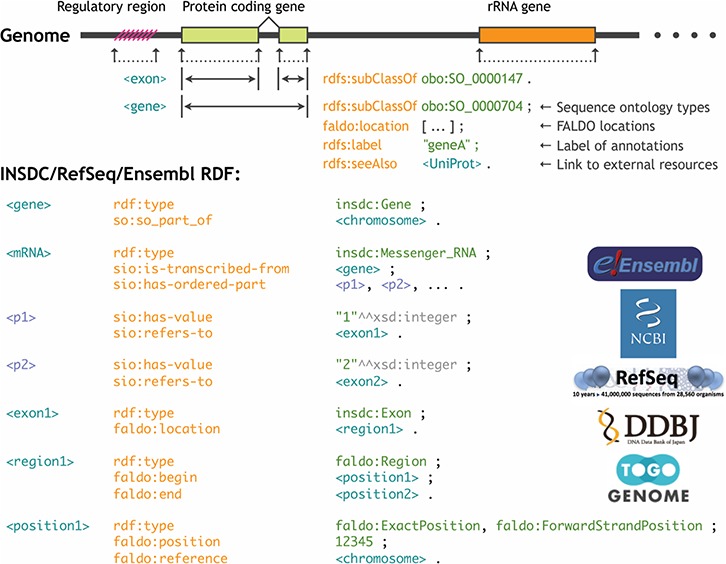
Standardization of the genome annotation coordinate system by FALDO.

Any annotations related to genome regions, such as gene structures, regulatory regions, mutations and modifications, can be located using the genomic coordinate system. This information can be integrated by uniquely identifying the reference sequence, specifying the beginning and terminating positions of the region to which the annotation is attached, and designating the type of the annotation. However, if the ontologies and the RDF data model to describe these feature locations are not standardized, a query for one genome database cannot be interoperable for the another even if the genome annotations are provided in RDF. For this reason, during the BioHackathon 2013 (http://2013.biohackathon.org/) (14) and the RDF summit (https://github.com/dbcls/rdfsummit) coding events, we developed the Feature Annotation Location Description Ontology (FALDO) (15) along with the UniProt, Ensembl, INSDC (DDBJ) and TogoGenome groups (Figure 5). We also developed the JBrowse genome browser version 1.10.0 (16) to implement a SPARQL query for acquiring and visualizing the annotations expressed using FALDO. Traditionally, several standards, such as GFF (http://gmod.org/wiki/GFF3) and TrackHubs (17), have been developed to attach the annotations to the genome coordinates. The same can be achieved in RDF using FALDO along with some other major ontologies such as the Sequence Ontology (SO) (18) and the Semanticscience Integrated Ontology (SIO) (19). Therefore, it is possible to construct a genome browser that can be of practical application while ensuring compatibility of the annotation information among the genome data sets represented in RDF.

### TogoGenome data sets

**Figure 6:**
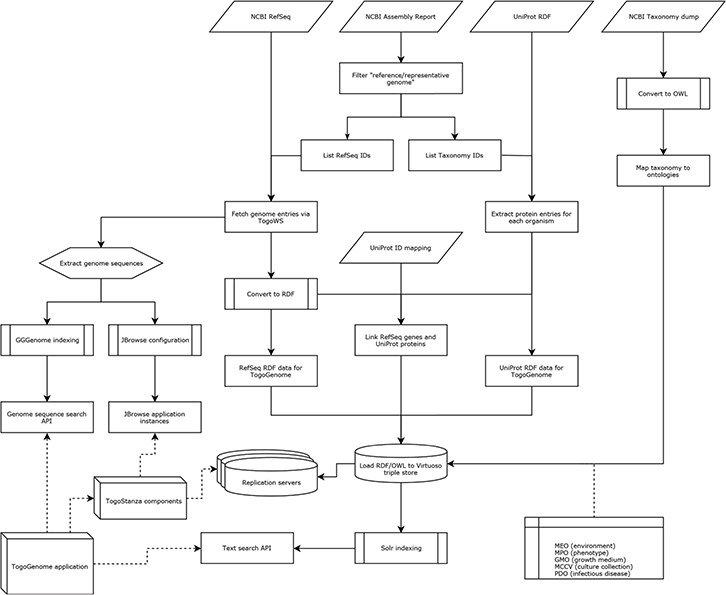
Procedure of data integration in TogoGenome.

On the basis of the above standardization, we developed RDF data sets and ontologies that are integrated with the public resources in case of TogoGenome (Figure 6).

#### Complete genomes

We selected the ‘reference genome’ and ‘representative genome’ entries from the NCBI assembly report and further extracted RefSeq and Taxonomy identifiers.

#### Genome annotations

We retrieved the NCBI RefSeq entries, including entire chromosome sequences, via the TogoWS service (20). Further, each entry was converted to RDF using an in-house developed converter, which is based on the BioRuby library (21) and represents the feature locations using FALDO. To semantically describe the types of annotations, we developed and incorporated the INSDC-annotated sequence ontology (Table 2) along with the taxonomy ontology described below. The converter is publicly available (Table 2), which is used to publish the RDF version of the INSDC entries from DDBJ (2), and is hosted at the NBDC RDF portal (Table 2).

#### Genome sequences

We extracted the genome sequences from the RefSeq entries and further indexed them for the JBrowse genome browser and the GGGenome sequence search service.

#### Taxonomy

We obtained a taxonomy dump from NCBI that contained all the species that were recorded in the INSDC sequence archive and their taxonomic hierarchies. Further, using the in-house developed converter (Table 2), we converted the dump to a Web Ontology Language (OWL) ontology file. The resulting ontology is publicly available (Table 2) and is used in the INSDC (DDBJ) RDF export.

**Table 2:** Availability of in-house developed converters and ontologies

**RDF data, ontologies and converters**	**URL**
INSDC RDF hosted at the NBDC RDF Portal	https://integbio.jp/rdf/
INSDC annotated sequence ontology	http://ddbj.nig.ac.jp/ontologies/nucleotide/
INSDC/RefSeq record to RDF converter	https://github.com/dbcls/rdfsummit/tree/master/insdc2ttl/
INSDC taxonomy ontology	http://ddbj.nig.ac.jp/ontologies/taxonomy/
NCBI taxonomy to INSDC taxonomy converter	https://github.com/dbcls/rdfsummit/tree/master/taxdump2owl/
MPO	https://bioportal.bioontology.org/ontologies/MPO
MEO	https://bioportal.bioontology.org/ontologies/MEO
MCCV	https://bioportal.bioontology.org/ontologies/MCCV
GMO	https://bioportal.bioontology.org/ontologies/GMO
PDO	https://bioportal.bioontology.org/ontologies/PDO

#### Protein information

We obtained the UniProt RDF files and extracted protein entries belonging to the species with complete genomes. Meanwhile, the correspondence between genes in the RefSeq entry and the UniProt protein entries was identified by using the UniProt’s ID-mapping file. Technically, it is possible to directly use the UniProt SPARQL endpoint; however, the performance of the SPARQL federated query was not satisfactory for our purpose and we only needed a subset of the entire UniProt database; we imported a portion of UniProt data of necessity into the TogoGenome.

#### In-house developed ontologies

We developed the Microbial Phenotype Ontology (MPO) for microbial phenotypes, Metagenome and Microbes Environmental Ontology (MEO) for habitat environments, Microbial Culture Collection Vocabulary (MCCV) for culture collections, Growth Medium Ontology (GMO) for growth media and Pathogenic Disease Ontology (PDO) for infectious diseases (Table 2). These ontologies were mapped onto the taxonomy ontology.

#### Other ontologies

We used FALDO for the annotation coordinates, SO- and INSDC-annotated sequence ontology for the types of annotated regions and GO for gene functions along with the common ontologies such as the SIO, Dublin Core terms, Simple Knowledge Organization System and so on.

As of June 2018, TogoGenome has integrated 7065 complete genome sequences of 2196 organisms (212 eukaryotes), which include 10 843 971 genes (4 070 521 eukaryotic genes), with the corresponding UniProt protein annotations. In total, ~6.3 billion triples of RDF data are stored and updated upon every RefSeq/UniProt release. The RDF database system, which is a triple store, that is currently being used is the Virtuoso open source version 7 (http://vos.openlinksw.com/) and it is scalable at least up to tens of billion triples in our experience. To improve the response of the SPARQL endpoint, the stored RDF data file in a loading instance is copied to the three backend Virtuoso instances (16 GB of each of the RAMs are allocated) for load balancing at the Nginx HTTP server layer. With this configuration, we can eliminate service downtime during the update procedure by sequentially updating and restarting these backend servers. This SPARQL endpoint is publicly available at http://togogenome.org/sparql for accepting customized queries from the users.

### Development of the TogoGenome

The TogoGenome application itself has been built using Ruby on Rails (https://rubyonrails.org/). Functions such as faceted search, comparative genomics and keyword and sequence searches, are implemented in this application layer. For the faceted search, we use several ontologies in combination such as (i) GO annotations imported from UniProt RDF for gene features, (ii) NCBI taxonomy that has been converted to OWL and released at DDBJ for organisms, (iii) MPO that has been developed for phenotypes and (iv) MEO that has been made for the habitat. The candidate ontology terms will be suggested while the keywords are being typed, and the user can traverse the hierarchy of ontologies to adjust the granularity of classification. To improve the performance of the faceted search, we calculated in advance the correspondences between the higher-level concepts in ontologies and genes that fall under the categories and stored the inferred relations at the time of updating data. Additionally, the combinations of the selected facets are stored in a user’s cookie, and the query results are cached as much as possible to improve the response time.

While SPARQL queries are suitable for semantic searches of interconnected objects in the RDF data sets, the efficiency of character string and regular expression searches is inefficient in most of the triple stores. In TogoGenome, we introduced the Apache Solr full-text search system for indexing the character strings such as names, descriptions and other text-based annotations of genes and organisms. However, identifying the page on which a searched term is displayed without tracing the connections between triples and pages was still difficult. To resolve this issue, we selected the targeted fields of the text searches in each TogoStanza and further indexed the strings and corresponding stanzas in pairs. For example, in the case of a gene report page, currently six stanzas contain literal annotations of a gene. We therefore create an index that contains a TogoGenome gene URI, a corresponding TogoStanza URI for the gene and a literal string contained in the TogoStanza. This indexing procedure is iterated over all genes of each organism stored in TogoGenome.

Similarly, searching for genomic regions that have a specific sequence with SPARQL is not efficient. Therefore, we indexed the genome sequences in the GGGenome system utilizing suffix array and FM-index (https://GGGenome.dbcls.jp/) and called the API to obtain the corresponding chromosome and its position. Using the specified sequence ID and location, genomic annotations around the region can be obtained by a SPARQL query using the FALDO.

### Development of the TogoStanza

Due to historical reasons, there are two branches of the TogoStanza development frameworks. TogoStanza was originally developed as a Ruby application but was later implemented also using JavaScript. Both of these branches are able to generate template files for SPARQL and HTML along with the files for metadata and supporting data.

The ‘Ruby version of the TogoStanza’ framework was released as a RubyGems’ package (https://rubygems.org/). Therefore, a user can install it via Ruby’s standard ‘gem’ command and further generate the TogoStanza template files using the installed ‘togostanza’ command. After customizing the templates and developing the query and visualization logic, the resulting TogoStanza can be deployed at the TogoStanza server and embedded into any web page as an IFRAME. Because the IFRAME encapsulates its content, other elements on a web page, even on a classical web browser, are not affected. However, due to the strict isolation of IFRAME, it is difficult to make a TogoStanza to interact with other TogoStanza even if both contain components that are embedded on the same page. Further, this version requires the TogoStanza process to keep running on the server while the SPARQL queries that are implemented inside the TogoStanza are executed on the server side. Therefore, a heavy load may be created while exhibiting exceeding accesses. This problem can be resolved in the following JavaScript version of TogoStanza.

The ‘JavaScript version of the TogoStanza’ module relies only on the standard web technologies, such as HTML, CSS, JavaScript, AJAX and SPARQL, and generates Web Components as a static HTML file. This eliminates the dependency on the server side where the SPARQL queries are made via an AJAX call directly from the user’s web browser to the public SPARQL endpoint. The results are rendered by the client browser. Using the Web Components technology, which encapsulates Document Object Model (DOM) as a shadow DOM, multiple TogoStanza can be embedded in a single DOM of a web page so that it is possible to implement components that react to an event that has been issued by another component upon a user’s interaction. The current drawback is that the state of a browser’s support, even while using a modern web browser, is not perfect for Web Components. Therefore, it will take a while for the transition from the Ruby version to the JavaScript version. Therefore, we provide a special Ruby version TogoStanza that wraps the JavaScript version as a temporal countermeasure.

## Discussion

By introducing the modularized architecture as TogoStanza, we and our collaborators were able to reduce the costs of mutually constructing new genome-related databases in TogoGenome, MicrobeDB.jp, MBGD (22) and CyanoBase. This exchange of the distributed resources could not be achieved by the existing monolithic genome database systems. The idea of providing reusable application components based on the web standard technology is a natural extension of the concept of the Semantic Web. In the Semantic Web, the RDF data stored in the distributed SPARQL endpoints are transparently accessible through the standard HTTP/HTTPS protocol unlike the data buried in the intranet database systems. Therefore, it is capable to use distributed heterogeneous data on a reciprocal basis. Additionally, RDF is scalable for the integration of heterogeneous data types without being bound by the database schema.

Traditionally, most of the genome databases are built on top of high-performance database engines such as a relational database (RDB) or key-value stores. We were unsure about the performance of emerging triple stores for RDF. Further, the original version of TogoGenome had been implemented using other triple stores or prior versions of Virtuoso and had not scaled enough in the beginning. However, the Virtuoso open source version 7, released in 2013, had a practical performance for our genome database by making tens of real-time SPARQL queries at once against billions of triples.

We have successfully demonstrated an RDF back-ended system with real-time SPARQL queries that can be used for a large-scale genome database. Meanwhile, we observed that triple stores were not efficient for text searches. However, this is not necessarily a defect of the Semantic Web system. Even while using RDBs or other NoSQL databases, it is usual to prepare a text search engine to perform keyword searches and an external application, such as BLAST, to perform sequence searches.

Traditional databases required the users to parse a database entry to extract information, forcing the users to develop custom scripts with programming language-depended open source libraries, such as BioPerl (23), Biopython (24), BioJava (25) and BioRuby (21), before performing real bioinformatics analyses. For databases that do not publish flat file dumps, a web interface that can retrieve the summarized information is often provided. However, the flexibility and granularity of information that can be obtained by a user are usually restricted by the capability of the provided APIs. In RDF, all the information is already parsed and semantically annotated. In case of SPARQL, especially with the ontologies and adaptable conditions, it is relatively straightforward to obtain any aggregated information by filtering data.

## Conclusions

We introduced a modularized architecture in the TogoGenome database that made the database developers to reuse the typical annotations of genes and organisms in other organism-specific or metagenome databases as embeddable TogoStanza components.

Because all the RDF data, a SPARQL endpoint, and TogoStanza components that are used in the TogoGenome application are publicly available, developers who intend to build another genome database will benefit from the usage of these resources to reduce the costs of application development and data management costs.

For future research, we plan to externalize SPARQL queries buried in the existing TogoStanza as REST APIs with the SPARQList (https://github.com/dbcls/sparqlist), so that advanced users can test and modify these queries for similar purposes with ease. Also, we have initiated the development of a human genome variation database through which the human subset of the TogoGenome can be reused and the information about mutations and genetic diseases can be enriched. Therefore, we are incorporating biomedical data sets in RDF and developing a corresponding set of TogoStanza.

## Notes

**Database URLs**: http://togogenome.org/ and http://togostanza.org/
